# Healthcare professionals’ experiences in using a patient-reported outcome tool (PRO-Pall) to identify symptoms and problems in palliative care: A mixed-methods study

**DOI:** 10.1017/S1478951525000483

**Published:** 2025-09-11

**Authors:** Tine Ikander, Heidi Bergenholtz, Henriette Sørensen, Marie Paine, Mikael Skytte, Ida Refer, Elise Christensen, Mette Raunkiaer, Cecilie Egholm

**Affiliations:** 1 REHPA, The Danish Knowledge Centre for Rehabilitation and Palliative Care, Odense University Hospital, Nyborg, Denmark; 2 Department of Clinical Medicine, University of Southern Denmark, Odense, Denmark; 3 Research Support Unit, Zealand University Hospital, Roskilde, Denmark; 4 Department of Surgery, Holbaek Hospital, Holbaek, Denmark; 5 Department of Respiratory Medicine and Infectious Diseases, Bispebjerg and Frederiksberg Hospital, Copenhagen, Denmark; 6 Holbæk Municipality, Holbæk, Denmark; 7 Center for Clinical Research and Prevention, Frederiksberg Hospital, Copenhagen, Denmark; 8 The Danish Health Data Authority, The National PRO-secretariat, Copenhagen, Denmark

**Keywords:** Palliative care, patient-reported outcome, mixed methods, general palliative care, patient-reported outcomes, heart disease, lung disease, kidney disease, cancer, health care professionals experiences

## Abstract

**Objectives:**

This study aimed to investigate healthcare professionals’ experiences with using the PRO Palliative Care questionnaire (PRO-Pall) to identify palliative care symptoms and problems in non-specialized palliative care settings among patients with heart, lung, and kidney disease, and cancer. The study also investigated the PRO-Pall’s potential to ensure further initiatives and care.

**Methods:**

A national, multicenter, observational study employing a mixed-methods approach. It includes quantitative analysis using an evaluation survey (*n* = 286) and qualitative analysis from workshops (*n* = 11). Quantitative data were analyzed descriptively, while qualitative data were analyzed thematically.

**Results:**

Quantitative and qualitative data were organized according to 3 a priori-defined themes: *Theme 1: Assessment of palliative symptoms, Theme 2: Support for dialogue*, and *Theme 3: Timely initiation of initiatives and care.* The evaluation survey and qualitative interviews with healthcare professionals indicated that it was valuable to use PRO-Pall in a non-specialist palliative context to screen for symptoms and problems, as well as to initiate actions. PRO-Pall helped to structure the dialogue and had a positive effect on the quality of the conversation.

**Significance of results:**

The findings highlight that it can be valuable to utilize the PRO-Pall in general palliative care settings for patients with heart, lung, or kidney diseases as well as cancer. When implementing PRO-Pall in practice, it is crucial to carefully consider the entire process, from administering the questionnaire to planning initiatives informed by patients’ PRO responses.

## Introduction

In recent decades, healthcare systems have acknowledged the importance of incorporating patients’ perspectives to ensure the delivery of high-quality services. This can be achieved in several ways, such as using patient-reported outcomes (PROs). PROs are health status reports directly from patients (U.S. Department of Health and Human Services FaDA, Center for Drug Evaluation and Research (CDER), Center for Biologics Evaluation and Research (CBER), Center for Devices and Radiological Health (CDRH) 2009), typically gathered through standardized generic or disease-specific questionnaires (PROMs). The systematic collection of patient-reported data holds promise for enhancing the delivery of palliative care (Churruca et al. 2021; Currow et al. 2008). Available research suggests that PROMs can play a role in palliative care by identifying symptoms and problems, improving patient assessment and care, and increasing patient satisfaction (Consolo et al. 2022; Dudgeon 2018; Easpaig et al. 2020; Etkind et al. 2015; Howell et al. 2015; Pinto et al. 2018; Ratzel et al. 2024; Skare et al. 2023). However, these studies have been conducted mainly in oncology or specialist palliative care settings.

Despite the growing body of evidence supporting the benefits of utilizing PROMs, systematic integration into standard clinical practice has not been realized (Basch 2017; Easpaig et al. 2020; Foster et al. 2018). Furthermore, filling out the PROMs cannot stand alone and requires follow-up by healthcare professionals, and if relevant, action based on the patient’s responses. However, to our knowledge, there has been limited investigation into healthcare professionals’ experiences in identifying symptoms and problems and how they take action in general (i.e. non-specialized) palliative care settings and in patients with diagnoses other than cancer. Etkind et al. report in a systematic review that using a PROM tool in specialized palliative care, primarily from oncology, allows more holistic care and more comprehensive recognition of symptoms, and the authors found evidence that healthcare professionals took more action when using a PROM (Etkind et al. 2015). A recent study by Müller et al. found that nurses and physicians in specialist palliative care identified unexpected symptoms using the PROM tool (Muller et al. 2023); however, it was not reported how healthcare professionals took action on the symptoms and problems reported by patients. Sandham et al. also express a need for studies on how PROs can benefit healthcare professionals as a decision support (Sandham et al. 2022).

In 2023, the Danish Health Data Authority launched a standardized PROM for non-specialized palliative care (hereafter “PRO-Pall”). This initiative aims to integrate PRO-Pall in general palliative care in primary care and hospitals for patients with heart, lung, and kidney diseases, and cancer (Egholm et al. 2023; PRO Palliation 2021). The PRO-Pall was developed to serve 3 main objectives: (1) to screen for symptoms and problems in general palliative care, (2) to support the dialogue between patients and healthcare professionals, and (3) to ensure timely initiation of initiatives and care as well as timely referral to specialist palliative care (Refer et al. 2022).

This study aimed to investigate healthcare professionals’ experiences using the PRO-Pall to identify symptoms and problems in non-specialized palliative care settings among patients with heart, lung, and kidney disease, and cancer. The study also investigated PRO-Pall’s potential to ensure further initiatives and care.

## Methods

This national, multicenter, observational study, employing mixed methods, was a part of a larger feasibility test of the PRO-Pall under the auspices of the Danish Health Data Authority (PRO-secretariat 2023; Refer et al. 2022). The feasibility test’s overall aim was to assess the experiences of using the PRO-Pall among both patients and healthcare professionals, and the present paper reports on the experiences of the healthcare professionals.

### The PRO-Pall

The core of the PRO-Pall is the European Organization for Research and Treatment of Cancer Quality of Life Questionnaire Core 15 Palliative Care (EORTC QLQ-C15-PAL) (Groenvold et al. 2006), which has been consistently used in Denmark among patients in specialist palliative care since 2012. Approximately 90% of patients referred to specialist palliative care are diagnosed with cancer. However, when developing the PRO-Pall for use in general palliative care, the target group was broadened to include patients with severe heart, lung, or kidney diseases in addition to cancer. This necessitated the inclusion of additional questions to reflect the broader target group and themes absent in the EORTC QLQ-C15-PAL. The PRO-Pall questionnaire consists of 24 items and was developed by a multidisciplinary group of palliative care experts and patients. Eight items addressing symptoms, social, and existential domains were added to the original EORTC-QLQ-C15-PAL. The new items included 3 items from the EORTC Library (EORTC) (edema, loneliness, and intimacy/sexual health), 5 newly developed items (sore and dry mouth, role change, emotional support, practical support, existential problems), and the “Write In Three Symptoms/Problems” item, allowing respondents to note any unmentioned symptoms or problems in free text (Rojas-Concha et al. 2020). An overview of the full PRO-Pall is available in Online Appendix 1. A description of the questionnaire’s development and structure, along with the content and user test report, is available (in Danish) on the Danish Health Data Authority’s website (PRO-secretariat).

### Setting and procedure for distribution of the PRO-Pall

The study was conducted in 3 municipalities, 3 general practices, a research clinic, and 8 departments across 5 hospitals. The 8 hospital departments covered a range of specialties, including cardiology (1), pulmonary medicine (3), nephrology (1), oncology (1), and surgery (2) (Refer et al. 2022).

The sites voluntarily participated in the PRO-Pall feasibility test, each testing it for about 6 months or until at least 50 patients responded. The first site started in November 2021 and the last site finished in October 2022. The test, commissioned by the Danish Health Data Authority, was coordinated by REHPA, the Danish Knowledge Centre for Rehabilitation and Palliative Care. Sites incorporated PRO-Pall into their usual care processes, using electronic methods or paper forms for data collection depending on local possibilities. The sites chose a time/trigger point for distribution of the questionnaire that suited local practices and the patients’ care processes, e.g. upon admission or when commencing ambulatory treatment. Patients eligible to respond to the PRO-Pall were adults with chronic or progressive life-threatening heart, lung, and kidney diseases, or cancer who were cognitively capable of completing the questionnaire.

### Mixed methods

The study draws upon a convergent parallel mixed-methods design inspired by Creswell and Clarke and involves the simultaneous collection of quantitative and qualitative data (Creswell and Clark 2017). In this design, the 2 data types are analyzed separately, with the ultimate goal of integrating and interpreting the independent results. This design was chosen as it allows researchers to approach a question from multiple perspectives. The convergent parallel design is especially valuable for exploring reasons and explanations behind quantitative outcomes. By comparing the quantitative and qualitative findings, we gained a comprehensive understanding of healthcare professionals’ experiences with PRO-Pall. The current study includes a quantitative, descriptive analysis of an evaluation survey as well as a deductive analysis of qualitative workshops. The convergent parallel design includes concurrent quantitative and qualitative phases, with data collected, analyzed, and presented separately for comparison. Results were reported by theme and synthesized in the discussion. In this study, Good Reporting of A Mixed-Methods Study (GRAMMS) was followed (Online Appendix 2) (O’Cathain et al. 2008).

### Data and analyses

Two methods – survey and workshops – were applied to assess healthcare professionals’ experiences of using the PRO-Pall to (1) screen for patients’ symptoms and problems, (2) support the dialogue between patients and healthcare professionals, and (3) ensure timely initiation of initiatives and care.

### Evaluation survey – quantitative data

The evaluation survey was developed by the Danish Health Data Authority to assess, from the perspective of healthcare professionals, PRO’s effectiveness to support identification of patient needs and care decisions as well as the quality of the conversation with the patient. The survey was paper-based with 7 questions and free-text fields (see Online Appendix 3 for the survey). Healthcare professionals at the participating sites were asked to complete an evaluation survey after each consultation where a patient had completed PRO-Pall. Data were subsequently entered into a REDCap database. Simple descriptive analyses were used to show the distribution of responses to each item (including missing responses), using the statistical software STATA. Given the aim of this study, only questions 1 through 5 were considered in the analyses.

### Evaluation workshops – qualitative data

Workshops were conducted at the pilot sites with healthcare professionals who had taken part in the feasibility test of the PRO-Pall as well as administrative staff who had supported its use to gain an in-depth understanding of their experiences using PRO-Pall in the consultation. All participants were appointed locally by a manager/project lead. An interview guide was applied, covering the a priori-selected topics of interest, e.g., perceived relevance of the PRO Pall and its effectiveness in terms of screening, supporting conversations, and care decisions (Online Appendix 4). The questions were based on experiences from previous PRO-development projects at the Danish Health Data Authority (Egholm et al. 2023). Each workshop had at least 2 interviewers/observers (E.H., M.R., and C.L.E., who all had experience in conducting interviews in groups, and I.F.R. as an observer). The sessions were audio-taped and transcribed. Data were analyzed deductively based on the 3 a priori-defined themes, derived from the objectives of the PRO-Pall. NVivo 1.4.1 was used for qualitative analysis. Data were analyzed by the 1st author, H.B., M.R., and C.L.E., with all other authors acting as critical peers.

### Mixed-methods synthesis

**Figure 1.**
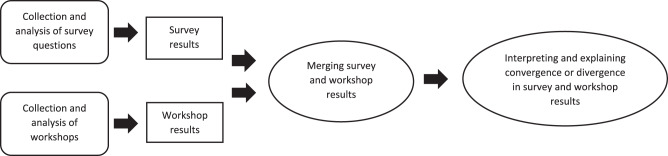
Illustration of the mixed-methods synthesis of data.

After the separate analysis of the quantitative and qualitative data, they were merged into 3 themes by linking the analyses of the qualitative data to the themes from the questionnaire (See Fig. 1). The themes will be presented in the result section.

### Ethical considerations

This research followed the principles of the Declaration of Helsinki (World Medical Association 2013), obtaining informed written consent from participants and adhering to national health science research requirements. The Scientific Ethics Committees for the Region of Southern Denmark determined that ethical notification was not applicable under Danish legislation (j.nr. 20222000–06). The study was registered with the Region of Southern Denmark’s data protection agency (j.nr. 22/4403), and data were securely stored in OPEN – Open Patient Data Explorative Network (OPEN 2024).

## Results

In total, 286 consultations including completed PRO-Pall assessments by patients were conducted. One hundred and two evaluation surveys were completed by a physician, 134 by nurses, 9 by both physicians and nurses, 10 by occupational therapists, 15 by physiotherapists, 7 by psychologists, and 4 by “other healthcare professionals,” e.g., psychologist students. For 5 surveys, the profession was not specified. A total of 42 healthcare professionals participated in 11 workshops, with 2–7 participants represented at each workshop. An overview of workshop participant characteristics is presented in Table 1.

**Table 1.** Number of workshops and participant characteristics

No. of workshops (total)	11
*Municipalities and general practices*^a^	4
*Hospitals*	6
*Research clinic*	1
Staff (total)	42 (100%)
*Physician*	5 (12%)
*Nurse*	27 (64%)
*Physiotherapist*	3 (7%)
*Administrative position*	7 (17%)
Gender (female)	40 (95%)
Years of experience with palliative care	Median 7 (mean 9.3), (min 0, max 30)
Years of experience in current position	Median 3.75 (mean 4.7), (min 0, max 16)

a General practices participated in the workshops together with the municipalities due to close cooperation in terms of providing palliative care to patients.

In the following, data are presented according to the 3 main themes: *Theme 1: Assessment of palliative symptoms, Theme 2: Support for dialogue*, and *Theme 3: Timely initiation of initiatives and care.* Each theme will be introduced with a presentation of the quantitative findings, followed by the findings from the qualitative data.

### Theme 1: Assessment of palliative symptoms

The theme covers whether healthcare professionals identified new symptoms and problems through the PRO-Pall that were not previously addressed and whether they felt any important questions were missing from the questionnaire.

In the evaluation survey, half of the respondents (48.6%) answered that they identified symptoms and problems among the patients through the PRO-Pall that were not already addressed (Table 2). Furthermore, 79% found that the PRO-Pall was sufficient to cover the assessment of palliative care symptoms and problems, whereas nearly 20% of the respondents lacked questions for a sufficient assessment of the patients’ symptoms and problems in palliative care. These findings were elaborated upon in the workshops. Reflecting the findings from the evaluation survey, some healthcare professionals communicated during workshop sessions about the identification of unmet symptoms and problems by using PRO-Pall. This was exemplified by a nurse: “What it has specifically done is actually with the dry mouth or oral discomfort; we haven’t really focused on that much, so it’s actually an eye-opener… similarly with edema, that there can be a lot of edema in the body that they wouldn’t mention themselves, and that we might not have thought about, but which is then mentioned because there’s a question about it” (Nurse, municipality). PRO-Pall thus helped draw attention to symptoms and problems that may have been overlooked previously. Furthermore, some healthcare professionals mentioned that family-related issues had become more apparent by using PRO-Pall, and found a need to talk about family matters among patients. A physician said: “I have had more conversations about relationship issues and sexual problems in these forms or in the conversations I’ve had here [utilizing PRO-Pall] than I have had in my entire medical career” (Physician, hospital). However, almost 50% in the evaluation survey indicated that they did not discover any new symptoms or issues by using PRO-Pall (Table 2). This experience was also stated during the workshops: “In terms of screening for palliative symptoms… I don’t think I encounter more issues than I’m used to in conversations – just the usual symptoms, generally speaking” (Nurse, Municipality).

**Table 2.** Results evaluation survey (*n* = 286)

Question^a^	Did you identify any patient needs through the questionnaire responses that were not already addressed?	Are you lacking any questions to assess the patient’s palliative care needs?	Did you or other professionals take any actions based on the responses?	Question^a^	To what extent did you utilize the patients’ responses during the dialogue?	Question^a^	In your opinion, how did the patient’s PRO response affect the quality of the conversation?
Response option, *n* (%)				Response option, *n* (%)		Response option, *n* (%)	
Yes	139 (48.6%)	57 (19.9%)	154 (53.9%)	1 (Not at all)	6 (2.3%)	1 (Very negative)	1 (0.4%)
No	142 (49.7%)	226 (79.0%)	124 (43.4%)	2	19 (7.1%)	2	3 (1.1%)
Missing	5 (1.7%)	3 (1.1%)	4 (1.4%)	3	77 (28.8%)	3	97 (33.9%)
Not relevant	–	–	4 (1.4%)	4	87 (32.6%)	4	93 (32.5%)
				5 (To a large extent)	78 (29.2)	5 (Very positive)	72 (25.2%)
				Missing	19 (6.6%)	Missing	20 (7%)

a The questions were translated from Danish solely for the purpose of presenting data in this article; however, this translation has not been validated.

Survey results showed that most healthcare professionals found the questionnaire sufficient with regard to doing a comprehensive assessment of the patient’s symptoms and problems. However, workshops revealed that certain questions were missing, particularly on gastrointestinal symptoms like, diarrhea, and problems with swallowing:

Well, I think it’s really good that they ask about constipation, but then they should also ask – I just think maybe they could rephrase it to general, you know, stomach issues, because we’re missing diarrhea in that, why only constipation? (Nurse, Municipality)

Furthermore, healthcare professionals lacked more questions to cover existential issues. A nurse said:

So, I think it would be good to have some more direct questions like … do you have any wishes for how you want to end your life, or do you have any questions about it… Have you thought about it, do you have any concerns? Is there anything you’d like support from us on, is there anything you’re unsure about, have you thought about death? (Nurse, Hospital)

Finally, a few healthcare professionals explained that the questionnaire did not need to be completely comprehensive: “No, because I think those questions lead you to elaborate on symptoms or issues, don’t they?” (Nurse, municipality). The healthcare professional points toward that the questions are designed to encourage deeper exploration and understanding of the patient’s condition.

### Theme 2: Support for dialogue

This theme encompasses results regarding whether healthcare professionals utilized patients’ PRO-Pall responses during the dialogue and if healthcare professionals experienced that integrating the patients’ PRO-Pall responses affected the quality of the dialogue.

In the evaluation survey, almost 90% responded that they utilized the patient’s response during the dialogue. Ninety-six percent responded that using the patients’ PRO-Pall responses affected the quality of the dialogue in a good way (Table 2). Like the quantitative results, the qualitative findings demonstrated that healthcare professionals utilized patients’ PRO-Pall responses. Healthcare professionals used patients’ PRO-Pall responses to structure the conversation: “The agenda is there, and then you can deviate a bit and add something, but you maintain the structure” (Nurse, municipality). There was also a perception about PRO-Pall being beneficial for the patients: “It provides a structure for the patients as well … they sort of know what’s coming, and sometimes it’s quite nice if you bring up something difficult” (Nurse, Hospital).

I also think for the patient – imagine experiencing – I’ve answered this chart at the hospital, and then a nurse comes out to me, she has seen the answers, she knows. That must be absolutely fantastic to experience, or to think, wow, you actually have that chart. I think they would also consider that to be really good quality, because it’s largely lacking, some sort of shared dialogue about it. (Nurse, Municipality)

In addition, the healthcare professionals used the PRO-Pall responses to quickly identify the symptoms and issues that patients found important to discuss:

The conversation based on PRO-Pall is fine in that you actually quickly pinpoint what you need to talk about…So in that way, it works really well as a framework for the conversation, so I would say that’s fine. (Nurse, Hospital)

Healthcare professionals believed the PRO-Pall tool improved the quality of patient conversations: “The PRO-Pall contributes to elevate the qualities of the conversation” (Nurse, Municipality)

### Theme 3: Timely initiation of initiatives and care

The theme encompasses results regarding whether healthcare professionals took any actions based on the patients’ PRO-Pall responses.

In the evaluation survey, almost 55% of the respondents reported that they or other healthcare professionals took initiatives based on the patient’s PRO-Pall responses (Table 2). Corresponding to the quantitative results, the qualitative results also indicated that actions were taken based on patients’ PRO-Pall responses, such as referrals to their general practitioner, municipality, psychologist, palliative care unit, or social nurse. Adjustments of medical treatment were, however less frequent:

So if we’re talking about medical treatment, not so much. I mean, there’s about a third of the patients where we’ve taken action, but it was through conversations or referrals to patient associations, to the municipality to get some practical help. So it might not be medical treatment, it’s something else. (Nurse, Hospital)

Some also mentioned that the patient had initiated actions based on their dialogue. Furthermore, several pointed out that the conversation itself is an action. A challenge that unfolded in the workshops was that healthcare professionals found it difficult to determine which actions should be taken based on symptoms. Often they experienced not knowing where to refer patients to due to either lack of knowledge of the options or lack of options:

1: It also requires knowing what kind of services one can offer.

2: Yes, that’s what I mean. And maybe they [the patients] would expect me to come up with an answer if they actually expressed that this is a problem, then they might expect me to come up with something, and right now I actually don’t know what I would offer right away. (Nurse, Municipality)

Intimacy-related issues also emerged as a topic where the healthcare professionals often struggled with delivering proper actions: “… if I were to receive an answer like, ‘yes, I really miss sex a lot.’ Then we really need to prepare some nurses for that because it won’t do any good if we just say, ‘oh, that’s too bad, I’ll just input that into the system’” (Nurse, Hospital).

An aspect that also became evident in the workshops was that a conversation’s outcomes are not predetermined but rather influenced by the specific individuals involved and the situation in which they are engaging: “The PRO-Pall is very profession-dependent and situation-dependent …so I’m thinking, what comes out of the conversation depends on who is talking to whom in which situation,” a municipality physician reported.

## Discussion

In the following section, the main findings within the 3 themes will be discussed. *1: Assessment of palliative symptoms, 2: Support for dialogue*, and *3: Better treatment.* The evaluation survey and interviews with healthcare professionals indicated that PRO-Pall is valuable in non-specialist palliative care for screening symptoms, initiating actions, and structuring dialogue, improving conversation quality.

### Assessment of palliative symptoms

The majority of the healthcare professionals did not miss any questions in the PRO-Pall tool, indicating that the tool overall is well-designed and adequate for the broad target group. However, workshops revealed that some participants wanted additional questions to better identify symptoms and problems. This highlights a gap between the content of the PRO-Pall and healthcare professionals’ needs, suggesting areas for improvement and also it points to a dilemma between generic schemes versus disease-specific schemes. The use of PRO-Pall facilitated the identification of previously unaddressed symptoms or problems for almost half of the healthcare professionals participating in the evaluation survey. This aligns with previous studies, which highlight PRO’s capacity for the identification of additional symptoms and problems throughout a wide range of clinical areas and cancer (Basch 2017; Burner-Fritsch et al. 2023; Gibbons et al. 2021; Sorensen et al. 2022). Similarly, Campbell et al. (2022) noted that PRO helped identify problems that may otherwise have been overlooked and emphasized its role in enabling tailored care (Campbell et al. 2022). Easpaig et al. further support these findings, reporting that PROMs were beneficial for healthcare professionals in a wide range of issues related to patient wellbeing (Easpaig et al. 2020). These issues included psychosocial and quality of life aspects, often overlooked compared to medical aspects (Easpaig et al. 2020). These findings suggest that while PRO tools like PRO-Pall can uncover overlooked symptoms, their effectiveness depends on question relevance and comprehensiveness. Continuous refinement, based on feedback from healthcare professionals and patients, is essential to meet evolving patient care.

### Support for dialogue

Our study showed that PRO-Pall supports dialogue with patients and enhances the quality of conversations from the perspective of a majority of the healthcare professionals. This improvement in communication can be regarded as a significant factor for both patients and healthcare professionals, as it ensures that patients’ symptoms and problems are clearly understood and addressed. Our finding aligns with other studies that highlight how PRO data can create a clear and focused framework for conversations (Easpaig et al. 2020; Ratzel et al. 2024) improving conversations between patients and healthcare professionals (der Willik Em et al. 2022; Etkind et al. 2015). The improved quality of conversations has several positive implications. First, it can increase patient satisfaction and trust in their treatment process (der Willik Em et al. 2022). When patients feel heard and understood, they are more likely to actively engage in their care and adhere to the recommended treatment plans. Second, it can reduce the risk of misunderstandings and misdiagnosis, as healthcare professionals gain a more detailed picture of the patient’s symptoms and concerns through systematic use of PROMs. At an organizational level, the implementation of PRO-Pall can also lead to more efficient use of resources. By structuring conversations and focusing on the most pressing issues, healthcare professionals can work more targeted and effectively (der Willik Em et al. 2022).

### Timely initiation of initiatives and care

Our study highlighted that a significant challenge for healthcare professionals was to determine the appropriate actions based on symptoms and problems reported by patients, often arising from a lack of knowledge or available referral options. This issue is consistent with Campbell et al. who reported that, in the use of PROMs in general, some healthcare providers found it difficult to act on patients’ PRO responses (Campbell et al. 2022). They expressed concerns about being expected to address all reported issues without having the necessary resources to manage them effectively (Campbell et al. 2022). Additionally, this concern was highlighted by Ito et al. (2023).

Further, studies report that clear guidelines on how to use PRO in practice and prioritize and address patients’ symptoms and problems could be beneficial (der Willik Em et al. 2022; Stover et al. 2015; Velikova et al. 2002). Easpaig et al. highlight the need for coordinated professional groups and clear role delineation to ensure effective follow-up on PRO responses (Easpaig et al. 2020). This emphasizes that systemic and organizational factors are crucial alongside individual knowledge and resources in effectively using PRO data. Enhancing the education and training of healthcare providers on the use of PRO data could improve their confidence and ability to act on the information provided through PROMs (Easpaig et al. 2020). This could involve developing training programs that cover not only the interpretation of PRO responses but also practical strategies for addressing the reported issues and ensuring that there are clear referral pathways and that healthcare providers are aware of the available resources.

### Strengths and limitations

In this study, we combined qualitative and quantitative data to investigate healthcare professionals’ experiences using the PRO-Pall to identify palliative care symptoms and problems in non-specialized palliative care settings among patients with heart, lung, and kidney disease, and cancer. The study also investigated the PRO-Pall’s potential to ensure further initiatives and care. Integrating these different data types was challenging due to the need to align the qualitative findings with the quantitative data. However, we find that the primary strength of this study is also the use of mixed methods, which involves analyzing data from multiple perspectives.

This increases the credibility and trustworthiness of the findings since we examined the research question from various angles, enhancing the overall validity and reliability of the study. A strength is that we include various types of sites (primary and secondary sectors) and a wide range of professionals. This ensures that we capture perspectives from a large part of the diverse group of stakeholders involved in non-specialized palliative care. A potential bias is that the sites were voluntary, which may indicate they have a special interest in the field, unlike other sites that may be less interested and/or have fewer resources. Therefore, the findings may not be representative.

## Conclusion

The evaluation survey and workshops with healthcare professionals indicated that it was valuable to use PRO-Pall in a non-specialist palliative care context to screen for symptoms and problems and initiate actions. PRO-Pall helped to structure the dialogue and had a positive effect on the quality of the conversation. However, when implementing PRO-Pall in general palliative care, it is crucial to carefully consider the entire process, from screening to planning initiatives informed by patients’ PRO responses.

## Supporting information

10.1017/S1478951525000483.sm001 Ikander et al. supplementary material Ikander et al. supplementary material

## Supplementary material

The supplementary material for this article can be found at https://doi.org/10.1017/S1478951525000483.
