# Supplementary material for: Healthcare professionals’ experiences in using a patient-reported outcome tool (PRO-Pall) to identify symptoms and problems in palliative care: A mixed-methods study
Source: Palliat Support Care. 2025 Sep 11;23:e159. doi: 10.1017/S1478951525000483 (PMC13166694; doi:10.1017/S1478951525000483)
Supplement: Ikander et al. supplementary material [file S1478951525000483sup001.zip › S1478951525000483sup001/Appendix 1. The PRO-Pall questionnaire.docx]

**Appendix 1**

An overview of the items in the PRO-Pall tool. The translation from Danish to English has been performed for the purpose of academic papers only, and the translation has not been psychometrically evaluated. The table was first presented in the article: Sørensen HD et al. Using a patient-reported outcome measure to assess physical, psychosocial and existential issues in COPD. J Clin Med 2024;13(20):6200. doi: 10.3390/jcm13206200.

| Item number | Wording | Response categories | Type of symptom/problem | Source |
| --- | --- | --- | --- | --- |
| 1 | Do you have difficulty going for a short walk outdoors? | Not at all  A little  Somewhat  A lot | Physical | EORTC-QLQ-C15 PAL |
| 2 | Do you have to lie in bed or sit in a chair during the day? | Not at all  A little  Somewhat  A lot | Physical | EORTC-QLQ-C15 PAL |
| 3 | Do you need help eating dressing, washing or going to the toilet? | Not at all  A little  Somewhat  A lot | Physical | EORTC-QLQ-C15 PAL |
| 4 | During the past week, were you short of breath? | Not at all  A little  Somewhat  A lot | Physical | EORTC-QLQ-C15 PAL |
| 5 | During the past week, have you had pain? | Not at all  A little  Somewhat  A lot | Physical | EORTC-QLQ-C15 PAL |
| 6 | During the past week, have you had trouble sleeping? | Not at all  A little  Somewhat  A lot | Physical | EORTC-QLQ-C15 PAL |
| 7 | During the past week, have you felt weak? | Not at all  A little  Somewhat  A lot | Physical | EORTC-QLQ-C15 PAL |
| 8 | During the past week, have you lacked appetite? | Not at all  A little  Somewhat  A lot | Physical | EORTC-QLQ-C15 PAL |
| 9 | During the past week, have you felt nauseous? | Not at all  A little  Somewhat  A lot | Physical | EORTC-QLQ-C15 PAL |
| 10 | During the past week, have you been constipated? | Not at all  A little  Somewhat  A lot | Physical | EORTC-QLQ-C15 PAL |
| 11 | During the past week, were you tired? | Not at all  A little  Somewhat  A lot | Physical | EORTC-QLQ-C15 PAL |
| 12 | During the past week, did pain interfere with your daily activities? | Not at all  A little  Somewhat  A lot | Physical | EORTC-QLQ-C15 PAL |
| 13 | During the past week, did you feel tense? | Not at all  A little  Somewhat  A lot | Psychosocial | EORTC-QLQ-C15 PAL |
| 14 | During the past week, did you feel depressed? | Not at all  A little  Somewhat  A lot | Psychosocial | EORTC-QLQ-C15 PAL |
| 15 | How would you rate your overall quality of life during the last week? | 7 point Likert scale ranging from ‘Very poor’ to ‘Excellent’ | Quality of life | EORTC-QLQ-C15 PAL |
| 16 | During the past week, did you have sore or dry mouth? | Not at all  A little  Somewhat  A lot | Physical | New item |
| 17 | During the past week, have you had swelling in any part of the body? | Not at all  A little  Somewhat  A lot | Physical | EORTC item library |
| 18 | During the past week, have you missed intimacy? (e.g., closeness, tenderness, sex) | Not at all  A little  Somewhat  A lot | Psychosocial | EORTC item library |
| 19 | During the past week, I felt lonely | Not at all  A little  Somewhat  A lot | Psychosocial | EORTC item library |
| 20 | Have you been worried about whether your role towards family or friends has changed? | Not at all  A little  Somewhat  Alot | Psychosocial | New item |
| 21 | Have you had thoughts about life or your situation that you need to talk about? | Not at all  A little  Somewhat  Alot | Existential | New item |
| 22 | Have the problems you have had in connection with your illness (such as financial, practical or personal) been taken care of? | Problems have been taken care of;  Problems have mostly been taken care of;  Problems have partly been taken care of;  Problems have by and large not been taken care of;  Problems have not at all been taken care of | Psychosocial | New item |
| 23 | Have you been able to share your feelings with your family or friends as much as you would like? | All the time  Most of the time  Sometimes  Rarely  Not at all | Psychosocial | New item |
| 24 | Have you had any other significant symptoms or problems not mentioned in the questions above? | No  Yes. Please list the most important (up to three) and indicate the extent to which you have had the symptoms or problems in the last week | Any | WISP |
