# Supplementary material for: Healthcare professionals’ experiences in using a patient-reported outcome tool (PRO-Pall) to identify symptoms and problems in palliative care: A mixed-methods study
Source: Palliat Support Care. 2025 Sep 11;23:e159. doi: 10.1017/S1478951525000483 (PMC13166694; doi:10.1017/S1478951525000483)
Supplement: Ikander et al. supplementary material [file S1478951525000483sup001.zip › S1478951525000483sup001/Appendix 2. GRAMMS Good Reporting of A Mixed Methods Study.docx]

**Appendix 2.** Good Reporting of A Mixed Methods Study (GRAMMS)

**Describe the justification for using a mixed methods approach to the research question:**

Page 6

**Describe the design in terms of the purpose, priority and sequence of methods**

Page 6-7

**Describe each method in terms of sampling, data collection and analysis**

Page 6-7

**Describe where integration has occurred, how it has occurred and who has participated in it**

Page 6-7

**Describe any limitation of one method associated with the present of the other method**

Page 17

**Describe any insights gained from mixing or integrating methods**

Page 17
