# Supplementary material for: Healthcare professionals’ experiences in using a patient-reported outcome tool (PRO-Pall) to identify symptoms and problems in palliative care: A mixed-methods study
Source: Palliat Support Care. 2025 Sep 11;23:e159. doi: 10.1017/S1478951525000483 (PMC13166694; doi:10.1017/S1478951525000483)
Supplement: Ikander et al. supplementary material [file S1478951525000483sup001.zip › S1478951525000483sup001/Appendix 3. Survey Questions.docx]

**Appendix 3. Survey Questions**

Translated by the authors for the purpose of this paper.

|  | **Question** | **Answer** |
| --- | --- | --- |
| 1. | Did you identify any patient needs through the questionnaire responses that were not already addressed? | Yes     No |
| 2. | Did you or other professionals take any actions based on the responses? | Yes     No     Not relevant |
| 3. | Are you lacking any questions to assess the patient’s palliative care needs? | Yes     No |
| 4.2 | To what extent did you utilize the patients’ responses during the dialogue? | 1 (Not at all)    2    3    4    5 (To a very large extent) |
| 5. | In your opinion, how did the patient's PRO response affect the quality of the conversation? | 1 (Very negative)  2  3  4  5 (Very positive) |
| 6. | Did the patient express a need to talk about symptoms/issues, based on the response? | 1 (Not at all)    2    3    4    5 (To a very large extent) |
| 7. | Did you experience any problems or challenges in the conversation due to the use of the patient's PRO responses | Ja     Nej |
