# Supplementary material for: Healthcare professionals’ experiences in using a patient-reported outcome tool (PRO-Pall) to identify symptoms and problems in palliative care: A mixed-methods study
Source: Palliat Support Care. 2025 Sep 11;23:e159. doi: 10.1017/S1478951525000483 (PMC13166694; doi:10.1017/S1478951525000483)
Supplement: Ikander et al. supplementary material [file S1478951525000483sup001.zip › S1478951525000483sup001/Appendix 4. PRO-pall interviewguide_english.docx]

# **Appendix 4: Workshop interview guide**

Please note: Translated from Danish to English for the purpose of this paper only, and not a validated translation.

**Session 1: Purpose of the questionnaire**

Have you experienced that the questionnaire has contributed to screening for palliative care needs? (*probing questions e.g. which needs, were these confirmed by patients, how (mechanisms), new needs not otherwise detected*).

Have you experienced actions initiated based on the needs identified by the questionnaire?

*(probing questions e.g. which type of actions being increased, do the questionnaire affect where in the process the action takes place)*

Have you experienced that the questionnaire has contributed to dialogue in the conversation with the patients?

*(probing questions e.g. changes in the quality of the conversation, the patients readiness and active engagement)*

**Session 2: Target group for the questionnaire**

Who did you test the questionnaire on?

With which patients did the questionnaire work well?

For which patients were there challenges? And why?

**Session 3: Use of the questionnaire**

How have you found the questionnaire to work in your everyday work with patients?

*(probing questions e.g. extra time needed, adjustments to workflow)*

Did using the answers present any challenges in the conversation?

How did the implementation of the questionnaire go?

*(probing questions e.g. what (did not) work well)*

**Session 4: Content of the questionnaire**

How do you feel about the amount of questions?

Are there any questions you find irrelevant?

*(probing questions e.g. which ones, why, how)*

Are there questions you find relevant but need to be customised?

*(probing questions e.g. which ones, why, suggestions for adaptation)*

Are there any questions you are missing?

*(probing questions e.g. which ones, why)*

Is there a match between the patient’s answers and your clinical judgement of the patient?

If no, is there anything that needs to be adjusted in the questionnaire?
